# A Successful Outcome Despite Delayed Intervention for Cauda Equina Syndrome in a Young Patient with a Posterior Epidural Disc Extrusion

**DOI:** 10.7759/cureus.4645

**Published:** 2019-05-11

**Authors:** Luke Mugge, Andrew Caras, William Miller, Mark Buehler, Azedine Medhkour

**Affiliations:** 1 Neurological Surgery, Inova Neuroscience and Spine Institute, Falls Church, USA; 2 Neurological Surgery, The University of Toledo Medical Center, Toledo, USA; 3 Surgery, The University of Toledo Medical Center, Toledo, USA; 4 Medical Education, The University of Toledo Medical Center, Toledo, USA

**Keywords:** epidural, disc extrusion, cauda equina syndrome

## Abstract

Epidural disc extrusion is extremely rare and may cause cauda equina syndrome. This is a surgical emergency and needs rapid decompression. Although cauda equina is commonly caused by disc herniation, this is an unusual presentation with epidural disc extrusion. We present a very rare case of Cauda Equina syndrome, resulting from an epidural disc extrusion at L3-L4 level. Patient care and progress notes were reviewed along with pre-, post-, and intra-operative radiological imaging. Here, a 19-year-old male with a past medical history of type I diabetes mellitus, fell asleep on a chair at home in an unusual position and was unable to walk on awakening. The patient developed progressive neurological deficits including bilateral foot drop along with bowel and bladder dysfunction. In addition, he experienced paresthesia and severe lower back pain unresponsive to steroids. Pre-operative magnetic resonance imaging (MRI) demonstrated a herniated disk epidurally with disc extrusion and mass effect and compression at the L3-L4 level, wrapping around the posterior aspect of the dura. A diagnosis of cauda equina syndrome was made and surgical decompression was performed. Using microsurgical technique and fluoroscopic guidance, a bilateral laminectomy of L3 was achieved with bilateral partial laminectomy of L4, with bilateral foraminotomy of L4. After removal of the lamina, a mass was immediately visualized in the posterior epidural space. Further dissection of the substance and following it posteriorly, identified the mass as a portion of the extruded disc. Post-operatively, the patient experienced rapid recovery. In conclusion, this case demonstrates that a disc extrusion can occur within the epidural space and can cause cauda equina syndrome. As this presentation is unusual, surgeons must be aware that they may encounter disc mass in unexpected locations, in a clinically delayed setting, long after the initial onset of symptoms.

## Introduction

Cauda equina syndrome (CES) is an uncommon, yet serious neurological condition, often best treated with immediate surgical intervention. From a mechanistic stand point, CES arises from compression of the lumbar and sacral nerve roots at or below the conus medularus, creating a myriad of symptoms ranging from focal sensory and motor deficits to dysuria and bladder incontinence. Surgical intervention for CES caused by disc herniation includes lumbar laminectomy and discectomy in order to relieve spinal cord compression and reestablished thecal space patency. Causes of CES are diverse as any foreign or local tissue found to intrude or extrude into the spinal canal is potentially capable of causing compression. While magnetic resonance imaging (MRI) remains the imaging modality of choice for the assessment of this condition, imaging may not yield wholly satisfactory results. A report by Derincek et al. demonstrates that migrated lumbar disc can appear as an abscess and thus tumors and other causes of CES must be considered in the differential when this condition is encountered [1]. A case by Talavera et al. similarly describes how radiographic findings for a disc fragment, which can cause serious conditions such as CES, are not clear and can appear as infection, tumors, or alternative pathologies, necessitating removal and pathological analysis for ultimate diagnosis [2]. A report by Sengoz et al. of an epidural lumbar disc fragment migration additionally suggests that radiological appearance is not specific enough to rule out alternative diagnoses and urgent surgical intervention is necessary to avoid the development of permanent neurological deficits [3]. Therefore, while the constellation of symptoms, which represent CES, are classic and reproducible, the attributable cause may not be clear and imaging may not provide satisfactory insight into the origin.

Here, we present a case of the lumbar level disc extrusion. The location of this epidural disc extrusion permitted the development of CES within this patient and is an unusual occurrence, under-reported within the literature. This case is further notable and distinct from the cases above which describe lumbar disc herniation, wherein the lumbar disc fragment migration from an alternative level. For our case, epidural disc extrusion was noted to be at the same level within the lumbar spine and to extend posteriorly and laterally, completely encompassing the thecal sac on the right side. We describe the clinical presentation and the successful operative intervention used to achieve decompression, providing a comprehensive discussion of the clinical presentation and ideal surgical intervention for this cause of CES in a very young patient.

## Case presentation

Case

**Figure 1:**
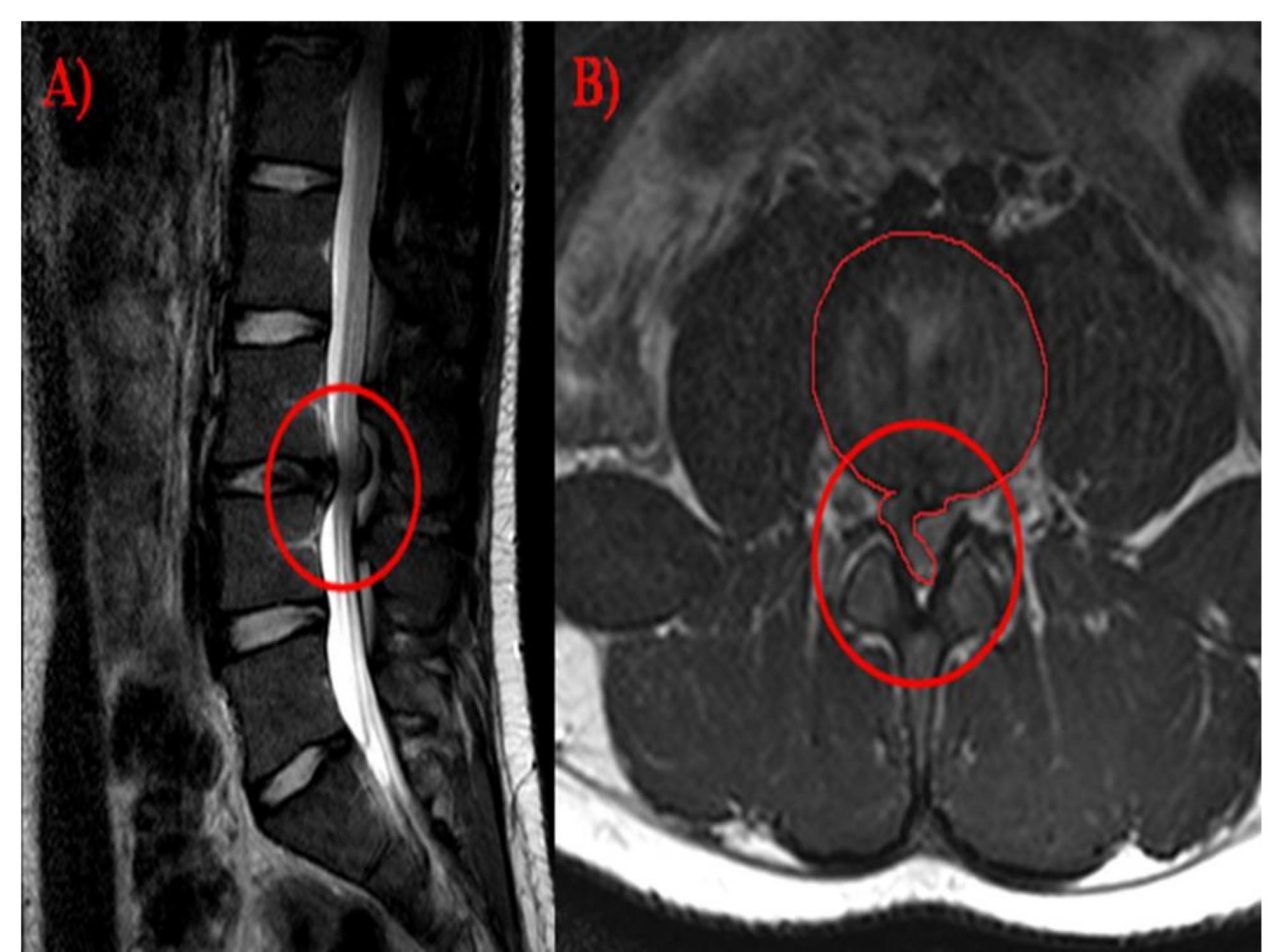
Pre-operative MRI (A) Sagittal T2 MRI showing disc extrusion at the L3-L4 disc space. (B) Axial T2 MRI showing the L3-L4 disc space with disc extrusion to the right and posterior to the thecal sac, resulting in mass effect to the left and compression of the cauda equina on the right. MRI: Magnetic resonance imaging

Our patient is a 19-year-old male who reports cigarette smoking ½ pack per day. Past medical history is significant for type I diabetes mellitus (DM). Our patient presented to the clinic with neurological deficits consisting of trouble walking and diffuse pain throughout his body when standing and considerable difficulty with balance. These symptoms began three months prior to his presentation in clinic when he fell asleep in an awkward position and, on awakening, found that he was unable to walk. There was severe lower back pain with initial paresthesia. He further indicated that he was having bowel dysfunction. On exam, it was noted that foot dorsiflexion and big toe extension was compromised. An intervertebral disc prolapse was suspected to have led to the development of cauda equina syndrome. Further, the patient was noted to have fatigue, change in appetite, muscle aches, muscle weakness, back pain, and swelling in his extremities. He indicates that he has restless legs due to the pain and migraines. It was decided that operative intervention would be beneficial, and radiological evaluation via MRI was undertaken with results presented in Figure 1.

Pre-operative findings

Congenitally shortened pedicles caused a congenital baseline spinal canal stenosis as seen in Figure 1. At the L3-L4 intervertebral disc level, there is a circumferential disc bulge with a superimposed right subarticular to right posterior midline epidural disc extrusion. On axial views, extruded disc material extends from the right subarticular location along the lateral epidural space, reaching the ligamentum and extending medially into the posterior epidural space. Disc material is seen on the sagittal view in the posterior epidural space with disc material contributing to severe spinal canal stenosis in both anterior and posterior epidural locations. There is mass effect on the traversing nerve roots of the cauda equina, displacing the nerve roots to the left of midline.

Operative procedure

**Figure 2:**
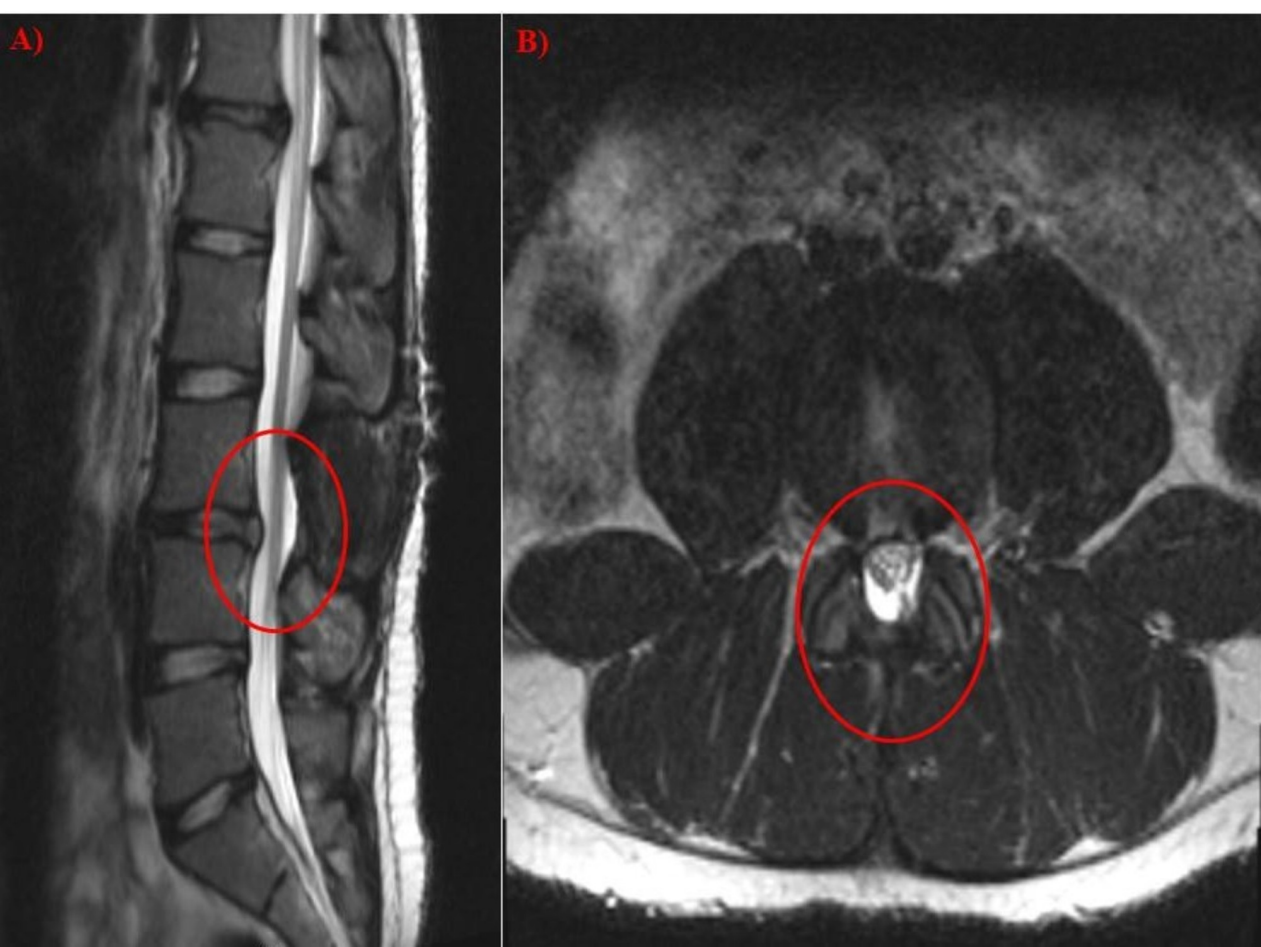
Post-operative MRI (A) Sagittal MRI T2 showing a laminectomy and removal of spinous process at the L3 level with removal of disc and restoration of the spinal canal space. (B) Axial T2 MRI of the L3-L4 disc space demonstrating the laminectomy and restoration of the thecal sack. MRI: Magnetic resonance imaging

Decompression seemed the best surgical intervention. In the operating room, the patient was placed in the prone position with chest rolls. Using microsurgical technique under both the microscope and fluoroscopic guidance, a bilateral laminectomy of L3 was achieved. Subsequently, a bilateral partial laminectomy of L4 was completed in addition to a bilateral foraminotomy of L4. Under the microscope, the dura was exposed and a large mass was identified and located directly posterior to the dura which was not fat or tumor. When this was followed to the right, this substance was revealed to be a portion of an extruded herniated disk, which was compressing the filum terminal in a tumor/mass effect-related manner. Careful dissection was carried out until the disk space was clearly exposed. After that, discectomy was accomplished at L3-L4 level. Foraminotomy of the L3-L4 level was completed bilaterally. Dura integrity was preserved throughout the process. Reconstruction was completed using an auto-fat graft. Musculature and subcutaneous fat was approximated. A JP drain was placed and the skin was closed. Post-operative imaging is demonstrated in Figure 2. Several aspects of this case are noteworthy from a surgical aspect. Given the young age of the patient, a complete foraminotomy on the affected side is inappropriate due to the rendered instability that it would cause. Further, the required instrumentation at such decompression would ultimately leave this patient with reduced range of motion. While this would not normally be of concern, the young age of the patient makes these interventions tenuous. As stated above, directly after the completion of the hemilaminectomy on the affected side, tissue of unknown origin was observed. Given that preoperative imaging revealed extruded disc at this level, it is likely this tissue was from the disk itself. However, the rare presentation and location of this disc extrusion, presenting within the epidural space, requires careful consideration of how to proceed next from an operative standpoint since the tissue, which is of an apparent etiology on imaging, may not be revealed to be of the same origin in actuality. When tissue, such as an extruded disc, is observed in an aberrant location, it is essential that careful tissue dissection and tracing to the inside of origin is established prior to complete tissue resection. Since a number of different tissues can present directly underneath the lamina, including infectious abscess, tumor, epidural lipomatosis, and others, early resection, removal, or debulking of the tissue may result in removal of tissue needed to identify the tissue based on origin and location. This necessitates occasionally unnecessary and delayed pathological analysis and complicates treatment. This case exemplifies successful surgical intervention wherein tissue was successfully dissected first posteriorly and then laterally to trace the tissue to the distal portion. This allowed for simultaneous identification of the tissue as benign in origin while successfully decompressing the cord, relieving mass effect.

Post-operative findings

Congenitally shortened pedicles, which caused a baseline congenital spinal canal stenosis, are consistent with pre-operative findings as seen in Figure 2. Lack of spinous process and lamina at the L3 level is consistent with interval laminectomy. There is evidence of removal of extruded disc material with restoration of nerve roots towards the midline and resolution of extrusion-induced mass effect. There is significant improvement of canal stenosis. The circumferential disc bulge previously noted at the L3-L4 level was significantly improved as compared to presentation on pre-operative studies. Together, it was noted the L3 disc extrusion visualized on pre-operative studies was significantly improved post resection with restoration of the nerve roots toward their expected central location.

Follow up

After surgery, the patient was found to be hypoglycemic as the only abnormality. He complained of only incisional pain. On exam, he was able to move all four extremities. He was noted to be hypertensive. The patient had severe pain on post-operative day 2. He was discharged on post-operative day 3. At the first post-operative visit, the patient was noted to walk without a walker. The patient indicated that his bowels were back to normal function. However he did have pain in the lumbar region that increased throughout the day. The patient had no observable acute neurological deficits.

## Discussion

This is an extremely rare case of CES resulting from a lumbar level epidural disc extrusion in a 19-year-old male. While CES is not uncommon, with symptoms including bilateral lower limb radiculopathy, hyperreflexia, bowel and bladder incontinence, and ambulatory instability, causes of this phenomenon are diverse in nature requiring diligent radiographic and intra-operative assessment. Regardless of the determined etiology, urgent surgical intervention to achieve decompression is best within the first 24-48 hours if permanent neurological deficits are to be avoided. Therefore, while having a precise diagnosis of the cause of CES is helpful in terms of establishing the most advantageous surgical approach, decompression by any approach is significantly better if it can be achieved within the early period rather than later.

CES commonly arises secondarily to compression of the cauda equina within the central canal resulting from a disc bulge or disc fragment migration. While disc involvement typically affects the level of its origin, producing corresponding symptoms, several reports have identified migrated disc fragments to cause CES when located within the epidural space. A case series by Turan et al. described nine patients who suffered from posterior epidural migration of a sequestered lumbar intervertebral disc fragment who subsequently required a discectomy via the posterior approach [4]. Tatli et al. similarly confirm that epidural disc fragment migration can cause severe neurological deficits in the form of either CES or conus medullaris syndrome [5].

By majority, disc herniation and nucleus pulposus extrusion into the thecal sac anteriorly is the most common cause of this syndrome. However, there are alternative causes. CES can rarely occur as a complication of a prior surgery, vascular pathologies, or alternative pathologies of the abdomen. A report by Maki et al. described a case of CES which occurred in the aftermath of a lumbar micro-discectomy, where the patient developed symptoms secondary to the shifting of the dural sac caused by ventral epidural venous plexus engorgement which developed as an operative complication [6]. Engamba et al. reported a case in which a patient presented with CES which resulted from the rupture of an underlying aortic aneurysm [7]. In a report by Singh et al., an 11-year-old boy was determined to have CES, which resulted from an underlying spinal hydatid cyst, which was initially thought to be an intradural extramedullary benign lesion [8]. Metastatic cancer has also been reported to cause CES. In a report by Solnes et al., a 32-year-old woman with metastatic breast cancer was noted to develop symptoms consistent with CES [9]. In addition to these, epidural disc fragment migration is also an uncommon, yet, significant cause of CES [10-13].

Posteriorly located lumbar disc extrusion as the cause of CES is unusual and under-reported within the literature. After completing a search of the literature, a minimal number of studies were identified, which explicitly discussed the presentation, radiographic findings, or surgical interventions recommended for lumbar disc extrusion resulting in CES. A case series by Zarrabian et al., which examined several patients with a lumbar disc extrusion from a radiological standpoint, concluded the diagnosis of disc extrusion is often challenging and overlooked, yet identified specific features including the presence of ventral and laterally located soft-tissue abnormalities which can stand as indicators for radiologists of a potential disc extrusion and aid in the application of an appropriate diagnosis [14]. In terms of a differential diagnosis, Williams et al. noted that a disc extrusion must be differentiated from either an epidural tumor or conversely anomalous root sheath [15]. Similar to our patient, a case report by Giri et al. described a case of CES caused by an epidural lumbar disc extrusion in a 16-year-old patient [16]. This case was complicated by the presence of an encapsulated hematoma within the epidural space, which was noted to be caused by the extruded disk itself.

Finally, when considering surgical intervention for this specific pathology of the lumbar spine, Tan et al. reported three successful cases of patients who suffered from CES who were successfully treated via the anterior approach with lumbar discectomy [17]. However, regardless of approach, early intervention for patients with complete or incomplete CES is necessary and associated with successful clinical outcomes with minimal neurological loss [18]. For our case, the presentation represented a challenge from a surgical standpoint. We achieved resolution of this particular presentation only through meticulous dissection of the disc along the posterior and right lateral margin of the thecal sac itself within the epidural space. This case highlights that the cause of CES may or may not be clear either on pre-operative imaging or intra-operatively and that disc fragments may extrude posteriorly, within the epidural space to cause this syndrome.

## Conclusions

We present a case of a 19-year-old with type I DM who developed CES as the results of a posterior disc extrusion within the epidural space. While it is unusual for this syndrome to develop over the course of three months, this case nonetheless demonstrates that decompression effectively restored neurological function no matter the duration of onset. Finally, given that this is an unusual cause of CES, we present the relevant imaging which describes pre-operative pathology and successful post-operative decompression for this condition.
